# Supplementary material for: Lenvatinib activates anti-tumor immunity by suppressing immunoinhibitory infiltrates in the tumor microenvironment of advanced hepatocellular carcinoma
Source: Commun Med (Lond). 2023 Oct 25;3:152. doi: 10.1038/s43856-023-00390-x (PMC10600115; doi:10.1038/s43856-023-00390-x)
Supplement: Supplementary file 1 — Description of Additional Supplementary Files [file 43856_2023_390_MOESM1_ESM.pdf]

Description of Additional Supplementary Files

File Name: Supplementary Data 1
Description: Annotations for the nCounter Tumor Signaling 360 Panel

File Name: Supplementary Data 2
Description: TCGA-TME classification platform

File Name: Supplementary Data 3
Description: The main source data for the figures
