# Supplementary material for: Lenvatinib activates anti-tumor immunity by suppressing immunoinhibitory infiltrates in the tumor microenvironment of advanced hepatocellular carcinoma
Source: Commun Med (Lond). 2023 Oct 25;3:152. doi: 10.1038/s43856-023-00390-x (PMC10600115; doi:10.1038/s43856-023-00390-x)
Supplement: Supplementary file 2 — Supplementary Information [file 43856_2023_390_MOESM2_ESM.pdf]

Supplementary Information

Lenvatinib activates anti-tumor immunity by suppressing immunoinhibitory infiltrates in the tumor microenvironment of advanced hepatocellular carcinoma

Masami Yamauchi*[1], Atsushi Ono[1], Kei Amioka[1], Yasutoshi Fujii[1], Hikaru Nakahara[1], Yuji Teraoka[1], Shinsuke Uchikawa[1], Hatsue Fujino[1], Takashi Nakahara[1], Eisuke Murakami[1], Wataru Okamoto[1], Daiki Miki[1], Tomokazu Kawaoka[1], Masataka Tsuge1, Michio Imamura[1], C. Nelson Hayes[1], Waka Ohishi[2], Takeshi Kishi[2], Mizuki Kimura[3], Natsumi Suzuki[3], Koji Arihiro[4], Hiroshi Aikata[1], Kazuaki Chayama*[5], and Shiro Oka*[1]

[1]*Department of Gastroenterology, Graduate School of Biomedical and Health Sciences, Hiroshima University, Hiroshima, Japan*

[2]*Department of Clinical Studies, Radiation Effects Research Foundation, Hiroshima, Japan*

[3]*Oncology department, Medical HQs, Eisai Co., Ltd., Tokyo, Japan*

[4]*Department of Anatomical Pathology, Hiroshima University Hospital, Hiroshima, Japan*

[5]*Collaborative Research Laboratory of Medical Innovation, Hiroshima University, Hiroshima, Japan*

**Corresponding authors: myamauchi@hiroshima-u.ac.jp; chayama@mba.ocn.ne.jp; oka4683@hiroshima-u.ac.jp*

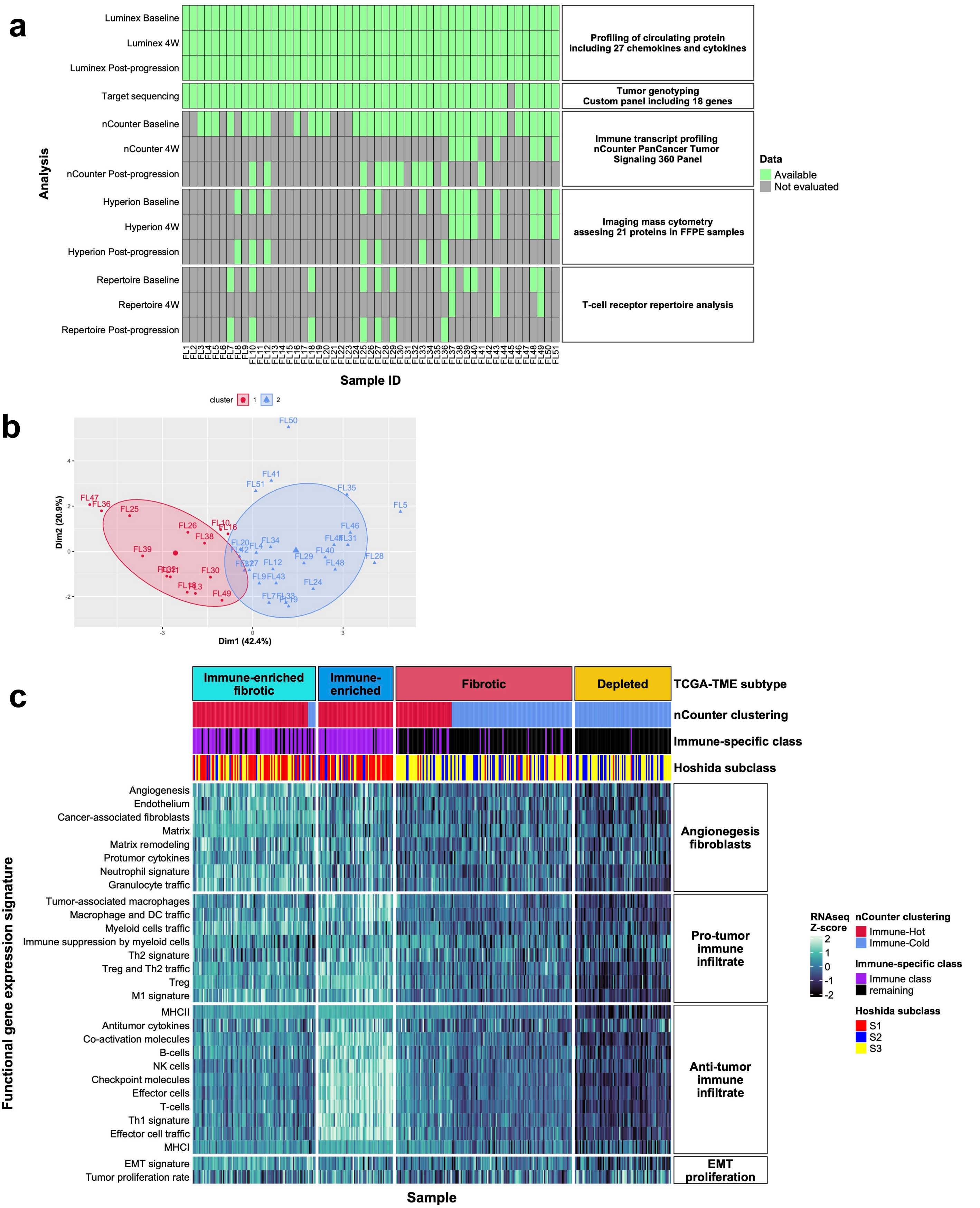

Supplementary Fig. 1: a, Correspondence table showing overview of timepoints, types of samples, and availability of samples for the present analysis. **b,** Reduced-dimension representation of annotated immune clusters. **c,** Heatmap of 371 TCGA hepatocellular carcinomas (TCGA-LIHC) classified into four TME subtypes based on clustering of the 29 functional gene expression signatures. Additional annotations over the heatmap represent immune subtypes calculated from the nCounter signatures used in the present analysis (Yamauchi M, 2023), Immune-specific class (Sia D, 2017[1]), and Hoshida S1-S2-S3 subclass (Hoshida Y, 2009[2]). Abbreviations: TCGA, The Cancer Genome Atlas; TME, tumor microenvironment; EMT, epithelial to mesenchymal transition.

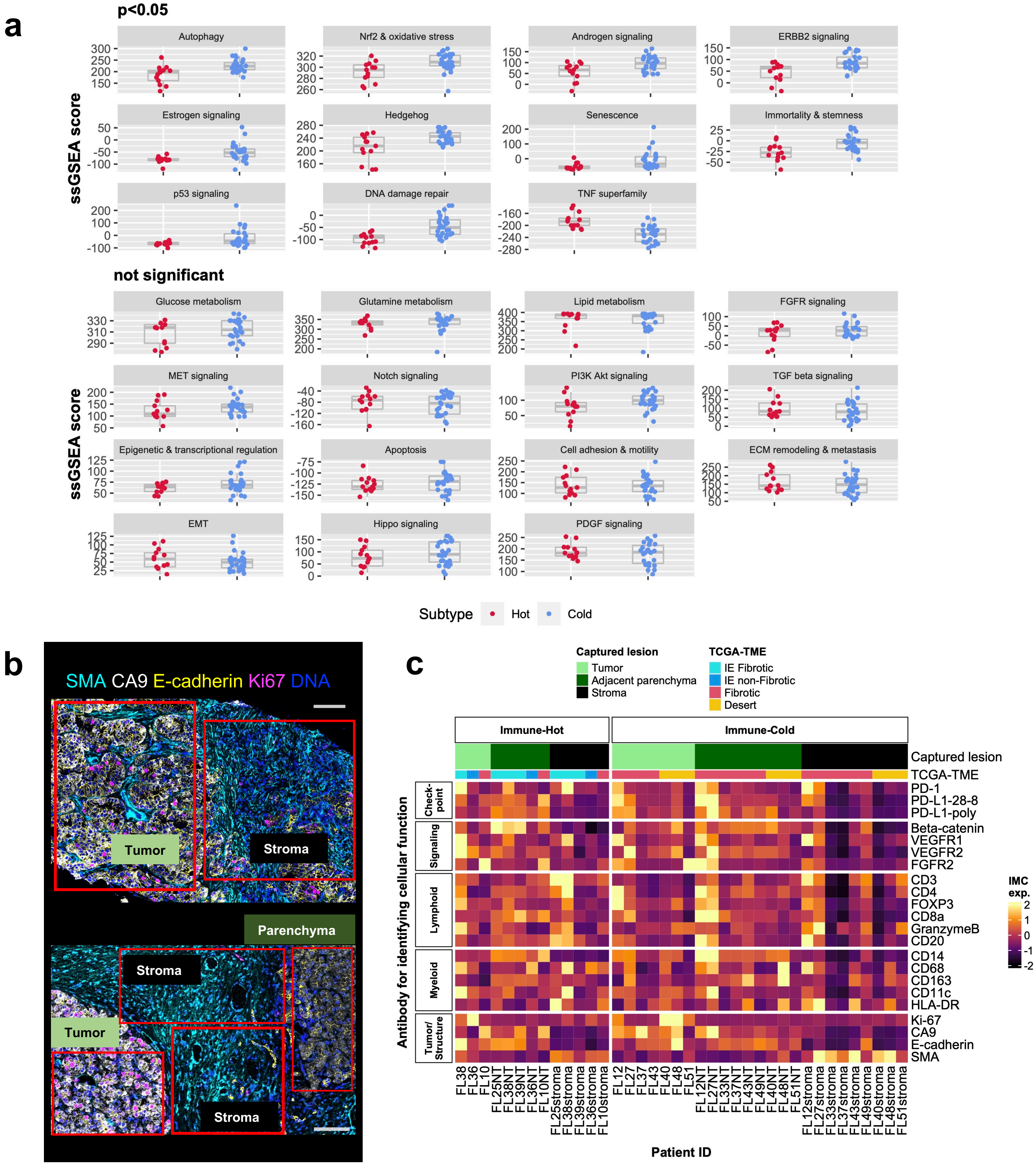

Supplementary Fig. 2: **a,** Box plots showing characteristics of each immune subtype regarding tumor signaling and tumor microenvironment. Each dot represents a single case. Boxes show the median (horizontal line), and the 25th and 75th percentiles. Whiskers indicate the 75th percentile plus 1.5 × interquartile range and the 25th percentile less 1.5 × interquartile range. Dots beyond whiskers are outliers. n=13 for Immune-Hot and n=26 for Immune-Cold. **b,** Images of ROI selection illustrating the method for calculating the expression of each imaging mass cytometry antibody. Scale bars, 100 µm. **c,** Heatmap showing expression of antibodies (signal intensity per square micrometer) stratified by immune subtypes and region in samples. Each expression is the mean value of two to five ROIs from the same patient. Abbreviations: ssGSEA, single-sample Gene Set Enrichment Analysis; TCGA, The Cancer Genome Atlas; TME, tumor microenvironment; IE, immune-enriched; IMC, imaging mass cytometry; exp., expression.

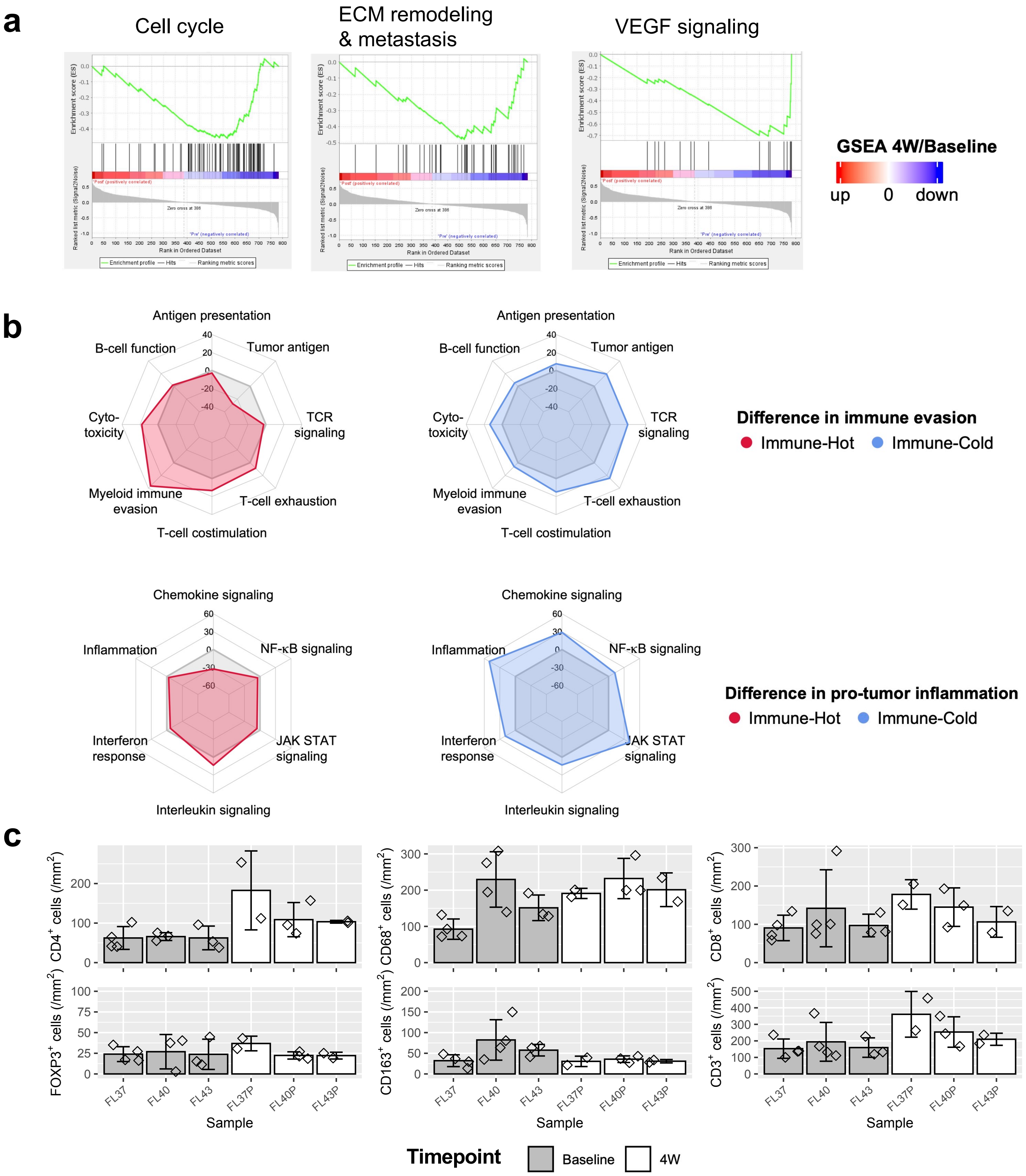

Supplementary Fig. 3: **a,** Representative enrichment plots. Inactivation of tumor signaling (cell cycle) and inactivation of tumor microenvironment (ECM remodeling & metastasis, VEGF signaling) in on-treatment samples. n=8 pairs (baseline and 4W) . **b,** Radar plots showing differences in scores for immune evasion and pro-tumor inflammation as calculated by nCounter signatures (ssGSEA values at 4W minus values at baseline). **c,** Bar plots showing absolute counts of CD4- or CD4- and FOXP3-positive cells, CD68- or CD68- and CD163-positive cells, and CD8- or CD8- and CD3-positive cells per square millimeter in the ROI captured from each tumor at baseline (grey) and during treatment (white). Each square dot represents a single case. Bars and error bars indicate the mean and its standard error. Abbreviations: GSEA, Gene Set Enrichment Analysis; ECM, extracellular matrix; VEGF, vascular endothelial growth factor; TCR, T-cell receptor; ssGSEA, single-sample Gene Set Enrichment Analysis; ROI, region of interest.

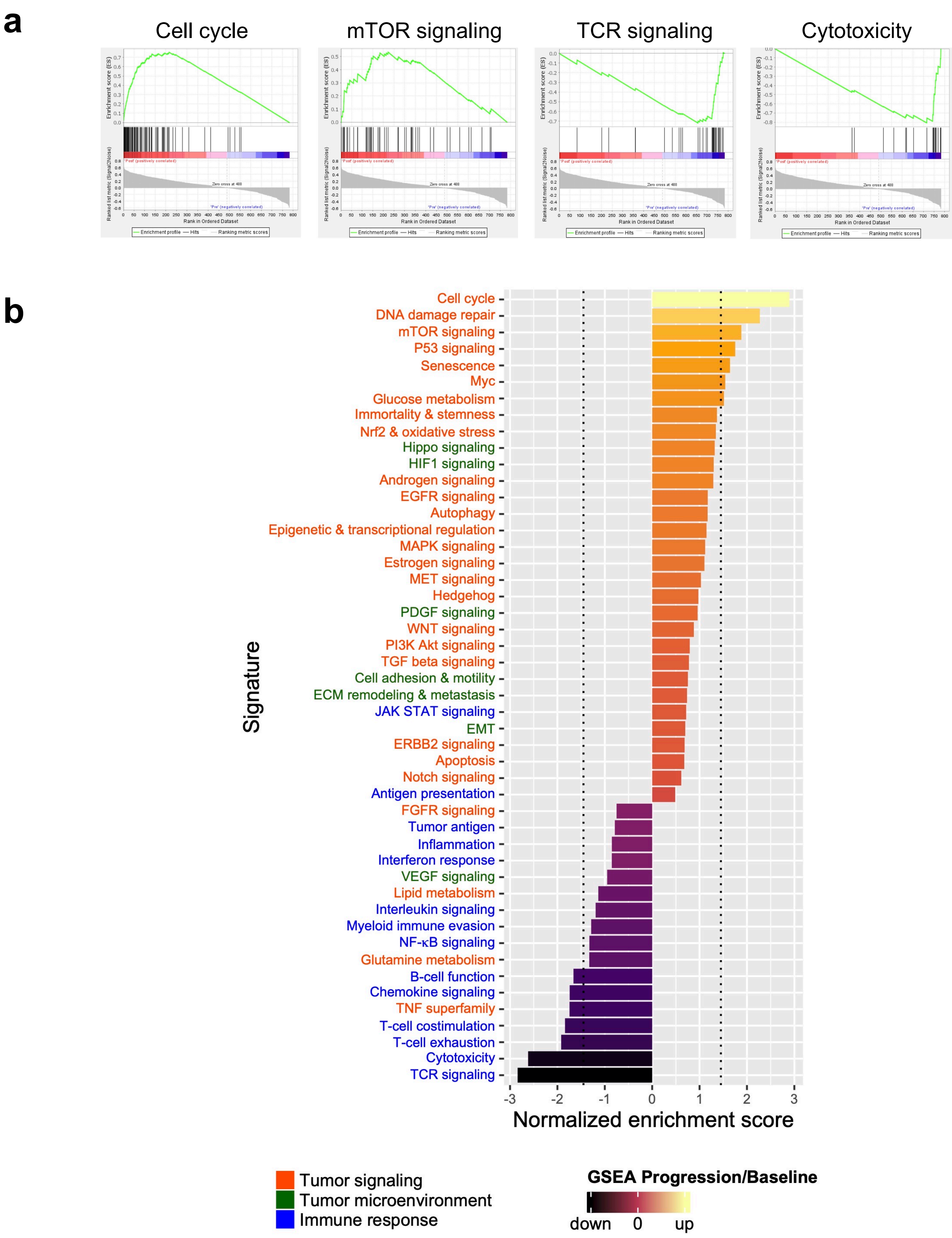

Supplementary Fig. 4: **a,** Representative enrichment plots. Activation of tumor signaling (cell cycle, mTOR signaling) and inactivation of immune response (TCR signaling, cytotoxicity) in post-treatment samples. **b,** Normalized enrichment scores of each signature in the nCounter Tumor Signaling Panel 360 (post-progression/pre-treatment). Dotted line indicates the statistical threshold for the significance of upregulation and downregulation in post-progression samples (P < 0.05). n=12 pairs (baseline and progression), (**a**, **b**). Abbreviations: TCR, T-cell receptor; GSEA, Gene Set Enrichment Analysis.

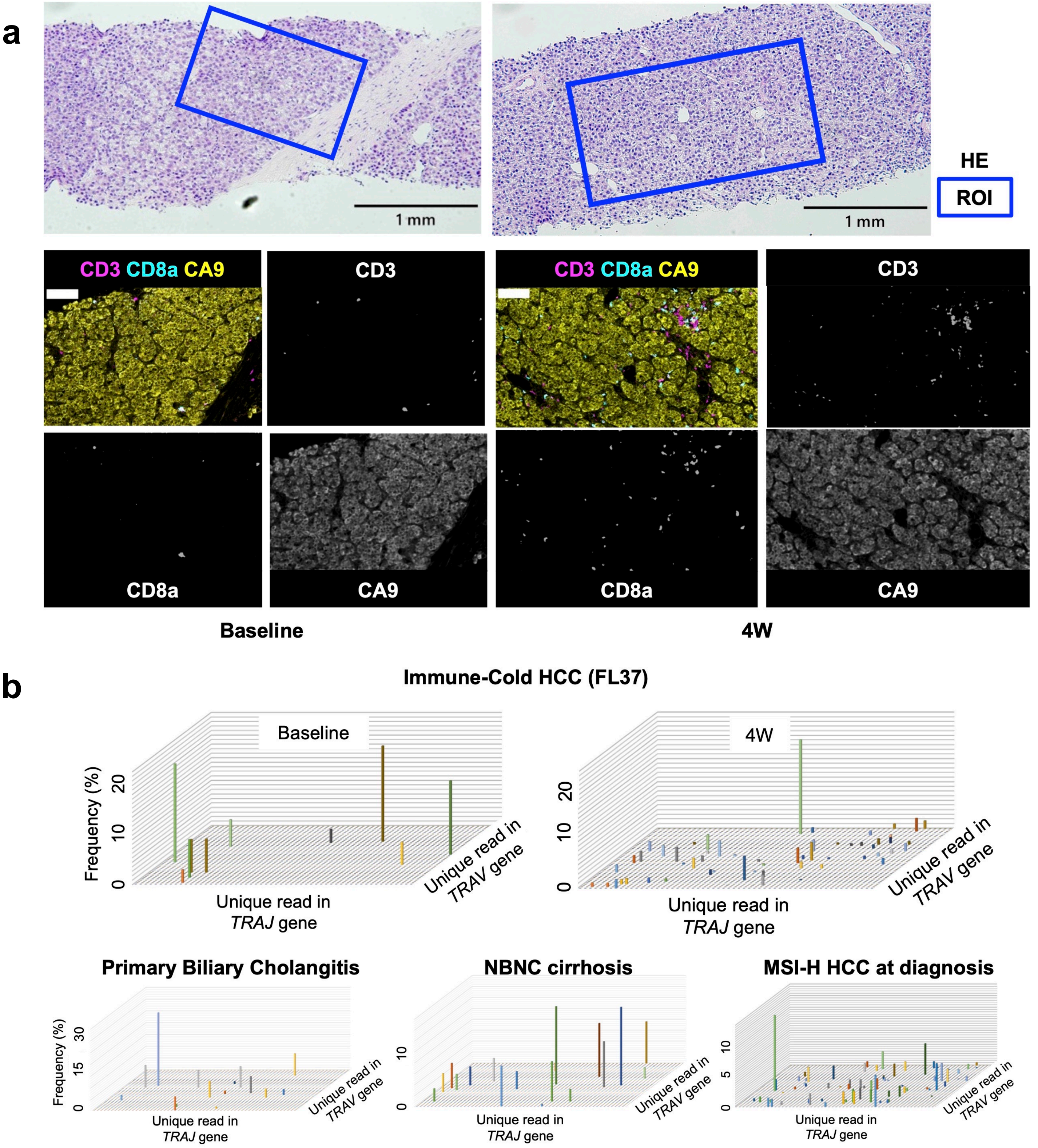

Supplementary Fig. 5: **a,** Mass cytometry images of HCC tumor at baseline and on-treatment showing overlay of CD3 (magenta), CD8 (cyan) and CA9 (yellow). Scale bars, 100 μm. Each color channel is split into grey-scale images in the lower three rows. HE images in the top row show corresponding laser-ablated areas (ROI). Scale bar, 1.0 mm. Images at 4 weeks representatively show the infiltration of CD8-positive lymphocytes into the viable tumor during treatment, even in cases that were Immune-Cold at baseline (FL40). **b,** Three-dimensional representation of *T-cell receptor alpha (TRA)* repertoires in Immune-Cold case (FL37) and controls. The x- and y-axes indicate the sequenced position of TRAJ and TRAV regions and the z-axis indicates frequencies of each TRA combination. Shannon-Weaver index H of primary biliary cholangitis, NBNC cirrhosis, and MSI-H HCC are 2.72, 3.08, and 4.09, respectively. Abbreviations: HE, hematoxylin and eosin stain; ROI, region of interest; NBNC cirrhosis, non-viral liver cirrhosis; MSI-H HCC, microsatellite instability-high hepatocellular carcinoma.

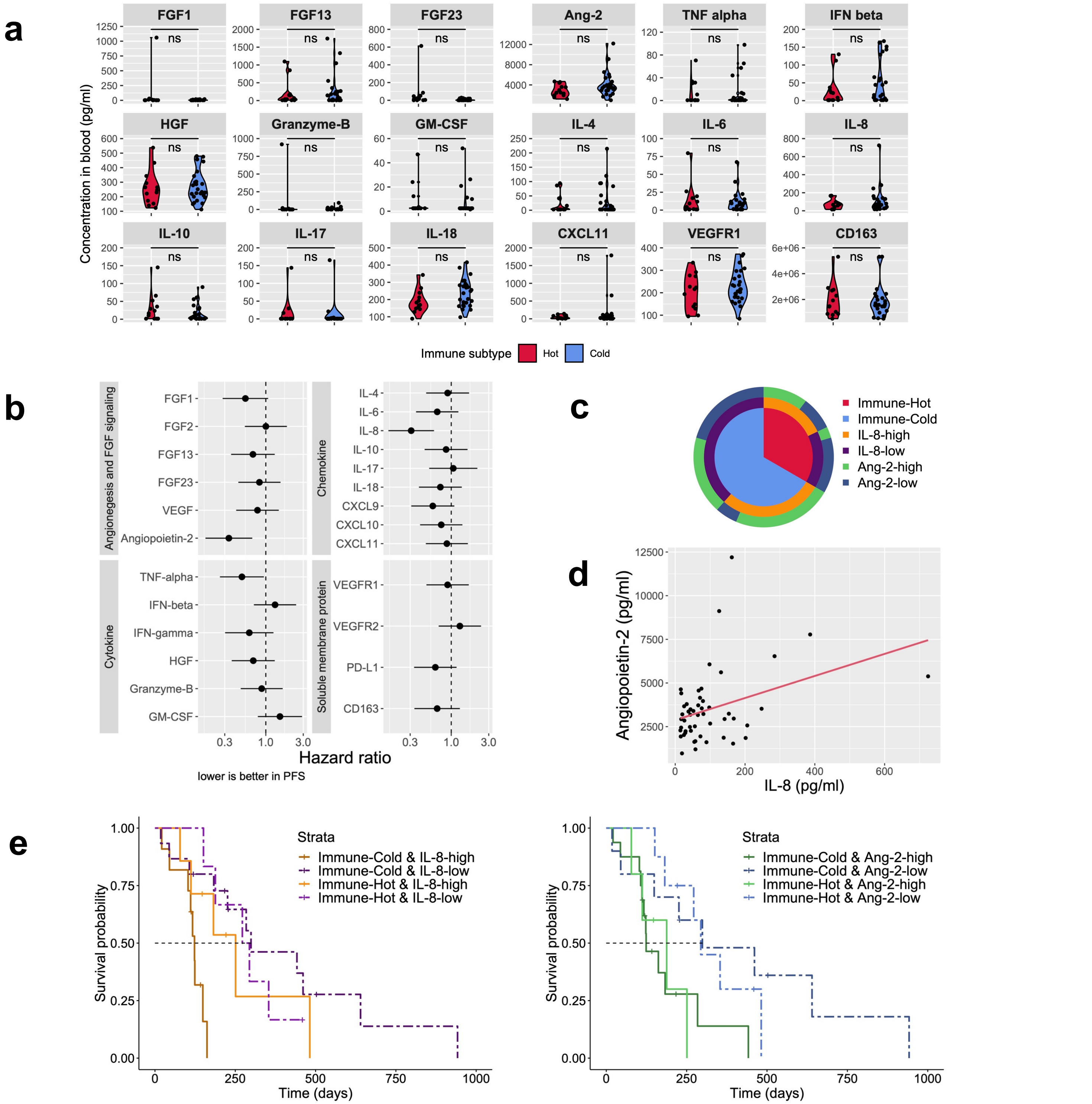

Supplementary Fig. 6: **a,** Levels of circulating proteins are not significantly different between the Immune-Hot subtype and the Immune Cold subtype except for CXCL10 (continued from **Fig. 5a**). Each dot represents a single case. **b,** Hazard ratios for disease progression according to baseline concentrations of circulating proteins in peripheral blood determined by the Luminex assay. Lower levels of IL-8 and Ang-2 were associated with better PFS based on Cox proportional hazard modeling, and hazard ratios were 0.31 (95%CI, 0.16–0.6) and 0.34 (95%CI, 0.17–0.67), respectively. **c,** Proportions of patients with elevated baseline IL-8 or Ang-2 levels in each immune subtype. **d,** Association between serum IL-8 and Ang-2 levels. Spearman's correlation coefficient for the association was 0.37 (95%CI, 0.10–0.58). Each dot represents a single case, and the red line shows the regression line. **e,** Higher levels of IL-8 and Ang-2 are associated with reduced survival benefit. PFS for Immune-Cold and IL-8-high (brown curve) and Immune-Hot and IL-8-high (orange curve) were 123 and 251 days, respectively. PFS for Immune-Cold and Ang-2-high (dark-green curve) and Immune-Hot and Ang-2-high (light-green curve) were 124 and 188 days, respectively. Dotted lines indicate the 50% probability of PFS. Median was used as a threshold value for each analyte in **a–e**. No multiplicity adjustments were made for any statistical analyses. n=13 for Immune-Hot and n=26 for Immune-Cold (**a**, **e**). n=51 (**b**, **d**). Abbreviations: ns, not significant; Ang-2, angiopoietin-2; PFS, progression-free survival; 95%CI, 95% confidence interval.

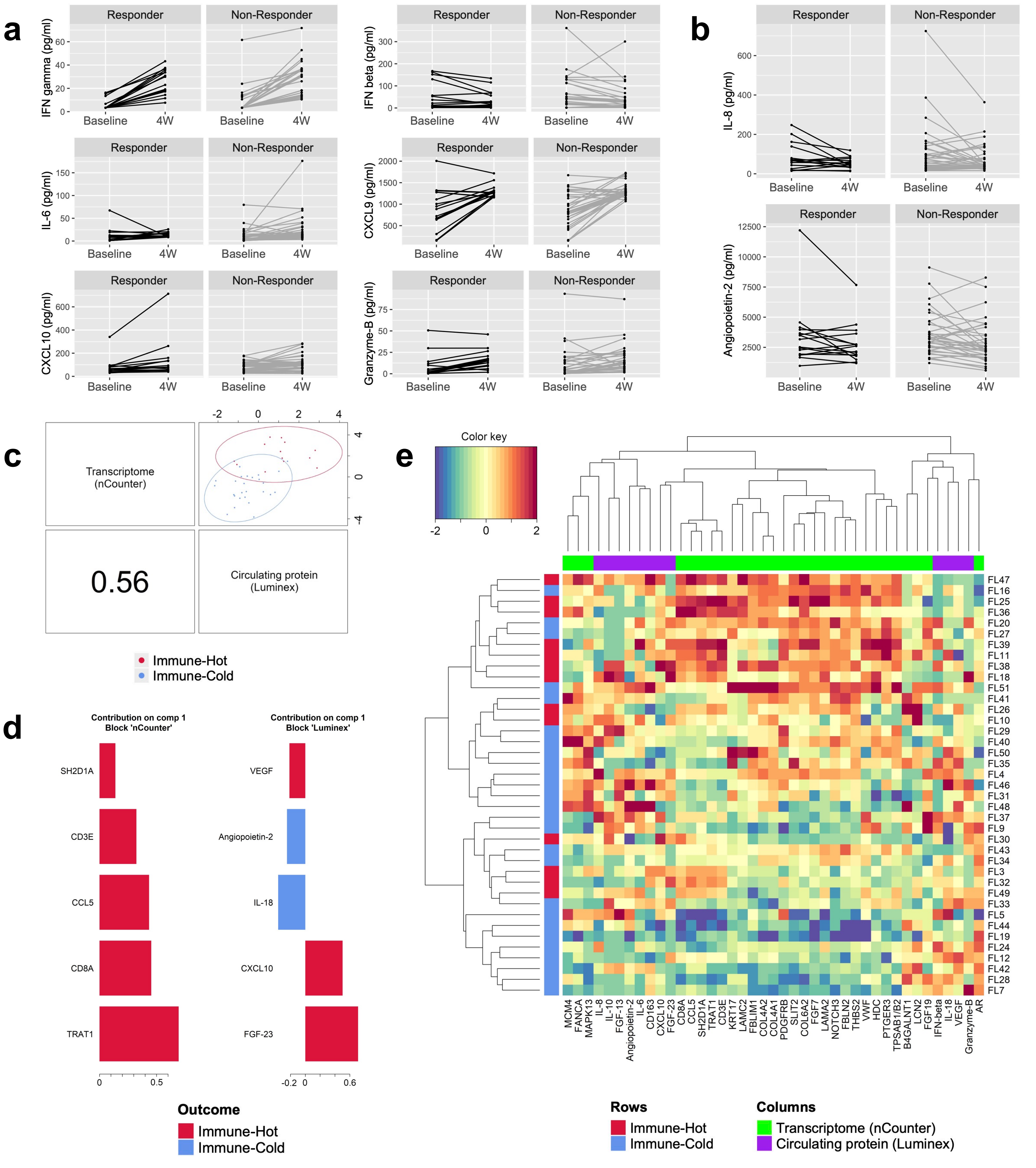

Supplementary Fig. 7: **a, b,** Line plots showing serial changes in serum concentrations of analytes shown in **Fig. 5c**, stratified by the response to lenvatinib therapy. The responder group (n=16) includes patients with RECIST CR, PR while the non-responder group (n=35) includes patients with SD, PD. **c,** Sample scatter plots showing the first component in each dataset analyzed thorough the mixOmics-Diablo platform. The Pearson correlation is 0.56. **d,** Lording bar plots showing the weight of original variables in omics data while producing latent components. **e,** Clustered image map confirming the contribution of selected features on the first component in columns. n=13 for Immune-Hot and n=26 for Immune-Cold (**c**, **d**, **e**). Abbreviations: RECIST, Response Evaluation Criteria in Solid Tumors; CR, complete response; PR, partial response; SD, stable disease; PD, progressive disease.

Supplementary Table 1 | List for eligibility

Key inclusion criteria	Key exclusion criteria
■ Age-lower limit: 20 years old or older<br>■ Age-upper limit: Not applicable<br>■ Sex: Male and female<br>■ Patients with advanced HCC in whom the first-line molecular targeted therapy or combined molecular targeted plus immune checkpoint inhibitor therapy begins within four weeks	■ Patients with allergy to lenvatinib, sorafenib, bevacizumab, atezolizumab, local anesthetics, or analgesics<br>■ Patients with severe ascites, severe renal failure, severe anemia, severe thrombocytopenia, jaundice, or coagulation disorder<br>■ Patients who need to take anticoagulants or anti-platelet drugs continuously<br>■ Patients with severe cognitive dysfunction or psychiatric disorders<br>■ Patients with severe heart failure<br>■ Pregnant or nursing mothers, women who desire to bear children

Supplementary Table 2 | Patient characteristics at baseline

Variable		Value
Total number of patients	N	51
Age - years	Median	72
	Range	46–88
Sex - n (%)	Male	44 (86)
	Female	7 (14)
Child-Pugh classification – n (%)	A	45 (88)
	B	6 (12)
Etiology of liver disease - n (%)	HBV	9 (18)
	HCV	16 (31)
	NBNC	26 (51)
BCLC stage - n (%)	B	27 (53)
	C	24 (47)
Extrahepatic metastasis - n (%)	+	18 (35)
	-	33 (65)
Previous therapy – n (%)	Surgery/ RFA	22 (43)
	TACE/ TAI	30 (59)
AFP – n (%)	< 400 ng/ml	31 (61)
	≧ 400 ng/ml	20 (39)

Abbreviations: HCC, hepatocellular carcinoma; HBV, hepatitis B virus; HCV, hepatitis C virus; NBNC, non-B, non-C hepatic disorder, including non-alcoholic fatty liver disease; BCLC stage, Barcelona Clinic Liver Cancer staging system; RFA, radiofrequency ablation; TACE, transarterial chemoembolization; TAI, transarterial chemoinfusion; AFP, alpha-fetoprotein.

Supplementary Table 3 | List of antibodies for imaging mass cytometry

Antibody name	Clone	Metal
**Alpha-SMA**	1A4	141Pr
**Beta-catenin**	D13A1	166Er
**E-cadherin**	24E10	158Gd
**Granzyme B**	EPR20129-217	167Er
**Ki-67**	B56	168Er
**FOXP3**	236A/E7	155Gd
**CA9**		163Dy
**CD3**	Polyclonal	170Er
**CD4**	EPR6855	156Gd
**CD8a**	C8/144B	162Dy
**CD11c**	Polyclonal	154Sm
**CD14**	EPR3653	144Nd
**CD20**	H1	161Dy
**CD68**	KP1	159Tb
**CD163**	EDHu-1	147Sm
**VEGFR1**	D-1	148Nd
**VEGFR2**	D-8	160Gd
**FGFR2**	1G3	153Eu
**PD-1**	EPR4877(2)	165Ho
**PD-L1**	28-8	150Nd
**PD-L1**	Polyclonal	170Er
**HLA-DR**	LN3	174Yb
**DNA intercalator**		191Ir
**DNA intercalator**		193Ir

Supplementary Table 4 | Result of sensitivity analysis for possible outliers

Circulating protein	Excluded value (ID, value at baseline)	P-value
**Immune subtype (Fig. 5a)**		
**CXCL10**	FL38, 340 pg/ml	0.060
**Change during treatment (Fig. 5c)**		
**CXCL10**	FL38, 340 pg/ml	<0.01
**IL-8**	FL4, 725 pg/ml	0.025
**IL-8**	FL4 and FL45, 387 pg/ml	0.063
**Angiopoietin-2**	FL48, 12197 pg/ml	0.008
**Angiopoietin-2**	FL48 and FL-46, 9118 pg/ml	0.013

Supplementary References

1. Sia, D., et al. Identification of an Immune-specific Class of Hepatocellular Carcinoma, Based on Molecular Features. Gastroenterology 153, 812-826 (2017).
2. Hoshida, Y., et al. Integrative transcriptome analysis reveals common molecular subclasses of human hepatocellular carcinoma. Cancer Res 69, 7385-92 (2009).
