# Supplementary material for: Lenvatinib activates anti-tumor immunity by suppressing immunoinhibitory infiltrates in the tumor microenvironment of advanced hepatocellular carcinoma
Source: Commun Med (Lond). 2023 Oct 25;3:152. doi: 10.1038/s43856-023-00390-x (PMC10600115; doi:10.1038/s43856-023-00390-x)
Supplement: Supplementary file 6 — Reporting Summary [file 43856_2023_390_MOESM6_ESM.pdf]

nature portfolio

Corresponding author(s): Masami Yamauchi

Last updated by author(s): Aug 17, 2023

Reporting Summary

Nature Portfolio wishes to improve the reproducibility of the work that we publish. This form provides structure for consistency and transparency in reporting. For further information on Nature Portfolio policies, see our Editorial Policies and the Editorial Policy Checklist.

Statistics

For all statistical analyses, confirm that the following items are present in the figure legend, table legend, main text, or Methods section.

n/a	Confirmed	
☐	☒	The exact sample size (*n*) for each experimental group/condition, given as a discrete number and unit of measurement
☐	☒	A statement on whether measurements were taken from distinct samples or whether the same sample was measured repeatedly
☐	☒	The statistical test(s) used AND whether they are one- or two-sided<br>*Only common tests should be described solely by name; describe more complex techniques in the Methods section.*
☐	☒	A description of all covariates tested
☐	☒	A description of any assumptions or corrections, such as tests of normality and adjustment for multiple comparisons
☐	☒	A full description of the statistical parameters including central tendency (e.g. means) or other basic estimates (e.g. regression coefficient) AND variation (e.g. standard deviation) or associated estimates of uncertainty (e.g. confidence intervals)
☐	☒	For null hypothesis testing, the test statistic (e.g. *F*, *t*, *r*) with confidence intervals, effect sizes, degrees of freedom and *P* value noted<br>*Give P values as exact values whenever suitable.*
☒	☐	For Bayesian analysis, information on the choice of priors and Markov chain Monte Carlo settings
☒	☐	For hierarchical and complex designs, identification of the appropriate level for tests and full reporting of outcomes
☐	☒	Estimates of effect sizes (e.g. Cohen's *d*, Pearson's *r*), indicating how they were calculated

Our web collection on statistics for biologists contains articles on many of the points above.

Software and code

Policy information about availability of computer code

Data collection	The acquired imaging mass cytometry (IMC) data were processed using the Hyperion Imaging System (Fluidigm, US). IMC images were assessed and analyzed on MCD Viewer version 1.0.560.6 (Fluidigm). Analysis of IMC antibody-positive cells was performed automatically using binary images processed with ImageJ software version 2.3.0/1.53t49 .
Data analysis	Custom codes, which are all written in R language and used in the current study, are available in the Figshare repository, [https://doi.org/10.6084/m9.figshare.22215760.v2].

For manuscripts utilizing custom algorithms or software that are central to the research but not yet described in published literature, software must be made available to editors and reviewers. We strongly encourage code deposition in a community repository (e.g. GitHub). See the Nature Portfolio guidelines for submitting code & software for further information.

Data

Policy information about availability of data

All manuscripts must include a data availability statement. This statement should provide the following information, where applicable:

- Accession codes, unique identifiers, or web links for publicly available datasets
- A description of any restrictions on data availability
- For clinical datasets or third party data, please ensure that the statement adheres to our policy

The datasets generated during the current study are available in the Figshare repository, [https://doi.org/10.6084/m9.figshare.22215760.v2]. All other data

supporting the results of this study are available from the corresponding author upon reasonable request.

Human research participants

Policy information about studies involving human research participants and Sex and Gender in Research.

Reporting on sex and gender	Sex assigned at birth was considered in this study.
Population characteristics	All patients characteristics at baseline are shown Supplementary Table 1.
Recruitment	The Key inclusion criteria was as follows; Patients with advanced HCC in whom the first-line molecular targeted therapy lenvatinib begins within four weeks. The key exclusion criteria were as follows; 1) Patients with allergy to lenvatinib, local anesthetics, or analgesics, 2)Patients with severe ascites, severe renal failure, severe anemia, severe thrombocytopenia, jaundice, or coagulation disorder, 3) patients who need to take anticoagulants or anti-platelet drugs continuously, 4) patients with severe cognitive dysfunction or psychiatric disorders, 5) patients with severe heart failure ,and 6) Pregnant or nursing mothers, woman who desire to bear children.
Ethics oversight	The Human Ethics Review Committees of Hiroshima University approved the study.

Note that full information on the approval of the study protocol must also be provided in the manuscript.

Field-specific reporting

Please select the one below that is the best fit for your research. If you are not sure, read the appropriate sections before making your selection.

☒ Life sciences ☐ Behavioural & social sciences ☐ Ecological, evolutionary & environmental sciences

For a reference copy of the document with all sections, see nature.com/documents/nr-reporting-summary-flat.pdf

Life sciences study design

All studies must disclose on these points even when the disclosure is negative.

Sample size	The sample size was based on sample availability at our institution.
Data exclusions	We included all patients and data into the analyses.
Replication	n/a. Samples of lenvatinib monotherapy are difficult to obtain in current clinical practice, as first-line treatment of hepatocellular carcinoma has shifted to combination immunotherapy.
Randomization	n/a. This trial was a single arm study confirming the effect of a single molecular targeted drug.
Blinding	Data on survival benefit and objective response were not blinded at the time of analyses. Immunological clustering of the tumor microenvironment at baseline was carefully performed in an unbiased manner based on unsupervised machine learning. Subsequent analyses were performed using these subtypes.

Reporting for specific materials, systems and methods

We require information from authors about some types of materials, experimental systems and methods used in many studies. Here, indicate whether each material, system or method listed is relevant to your study. If you are not sure if a list item applies to your research, read the appropriate section before selecting a response.

Materials & experimental systems

n/a	Involved in the study	
☐	☒	Antibodies
☒	☐	Eukaryotic cell lines
☒	☐	Palaeontology and archaeology
☒	☐	Animals and other organisms
☐	☒	Clinical data
☒	☐	Dual use research of concern

Methods

n/a	Involved in the study	
☒	☐	ChIP-seq
☒	☐	Flow cytometry
☒	☐	MRI-based neuroimaging

Antibodies

Antibodies used	All antibodies used for imaging mass cytometry (IMC) are described in the Supplementary Table 4.
Validation	Validation of the antibody's function was performed using archives of pathologically characterized (CD4, CD8, and FOXP3) neoplastic liver tissue from Hiroshima University Hospital (Nature Communications 2022,13:6481, [https://doi.org/10.1038/s41467-022-34249-x]); each concentration of IMC antibody was optimized by serial dilutions and different buffer solutions (pH 6 and 9).

Clinical data

Policy information about clinical studies

All manuscripts should comply with the ICMJE guidelines for publication of clinical research and a completed CONSORT checklist must be included with all submissions.

Clinical trial registration	The study was registered under UMIN000039887 and UMIN000044924 (observational study to assess the tumor microenvironment).
Study protocol	The study protocol will be available as Supplementary Information (in Japanese) if requested.
Data collection	Between April 2018 and March 2020 at Hiroshima University Hospital, 51 eligible patients were enrolled.
Outcomes	The main objective of the study UMIN000039887 was to assess the relationship between early histological change evoked by molecular-targeted therapy in HCC and subsequent survival outcomes. All the data including overall survival are now under analysis and will be published in the future.
